# Combination of psychotherapy and benzodiazepines versus either therapy alone for panic disorder: a systematic review

**DOI:** 10.1186/1471-244X-7-18

**Published:** 2007-05-14

**Authors:** Norio Watanabe, Rachel Churchill, Toshi A Furukawa

**Affiliations:** 1 Department of Psychiatry and Cognitive-Behavioral Medicine, Nagoya City University Graduate School of Medical Sciences, Nagoya, Japan; 2 Section of Evidence-Based Mental Health, Health Service and Population Research Department, Institute of Psychiatry, King's College London, University of London, UK

## Abstract

**Background:**

The efficacy of combined psychotherapy and benzodiazepine treatment for panic disorder is still unclear despite its widespread use. The present systematic review aims to examine its efficacy compared with either monotherapy alone.

**Methods:**

All randomised trials comparing combined psychotherapy and benzodiazepine for panic disorder with either therapy alone were identified by comprehensive electronic search on the Cochrane Registers, by checking references of relevant studies and of other reviews, and by contacting experts in the field. Two reviewers independently checked eligibility of trials, assessed quality of trials and extracted data from eligible trials using a standardized data extraction form. Our primary outcome was "response" defined by global judgement. Authors of the original trials were contacted for further unpublished data. Meta-analyses were undertaken synthesizing data from all relevant trials.

**Results:**

Only two studies, which compared the combination with behaviour (exposure) therapy, met our eligibility criteria. Both studies had a 16-week intervention. Unpublished data were retrieved for one study. The relative risk for response for the combination was 1.25 (95%CI: 0.78 to 2.03) during acute phase treatment, 0.78 (0.45 to 1.35) at the end of treatment, and 0.62 (0.36 to 1.07) at 6–12 months follow-up. Some secondary outcomes hinted at superiority of the combination during acute phase treatment.

One study was identified comparing the combination to benzodiazepine. The relative risk for response was 1.57 (0.83 to 2.98), 3.39 (1.03 to 11.21, statistically significant) and 2.31 (0.79 to 6.74) respectively. The superiority of the combination was observed on secondary outcomes at all the time points. No sub-group analyses were conducted due to the limited number of included trials.

**Conclusion:**

Unlike some narrative reviews in the literature, our systematic search established the paucity of high quality evidence for or against the combined psychotherapy plus benzodiazepine therapy for panic disorder. Based on limited available published and unpublished data, however, the combined therapy is probably to be recommended over benzodiazepine alone for panic disorder with agoraphobia. The combination might be superior to behaviour therapy alone during the acute phase, but afterwards this trend may be reversed. We know little from these trials about their adverse effects.

## Background

Panic disorder is characterized by the repeated occurrence of unexpected panic attacks, i.e. abrupt strong fears with anticipation of death, often accompanied by somatic symptoms such as palpitations, dyspnoea or faintness. Applying recent diagnostic criteria including DSM-III-R and DSM-IV, epidemiological studies have reported annual prevalence at 2.3% [1], 2.2% [2] and 2.7% [3]. One-third to one-half of patients with panic disorder in community samples is also diagnosed to have agoraphobia [4].

Two broad categories of treatment have been shown to be effective in treating panic disorder; one is pharmacotherapy including benzodiazepines and antidepressants, and the other is psychotherapy including behaviour therapy and cognitive behaviour therapy.

Recent guidelines recommended selective serotonin reuptake inhibitors (SSRIs) as the first-line pharmacological treatment and argued that its efficacy was comparable to psychotherapy alone [5]. However, the most recently published systematic review has shown that the combined antidepressant plus psychotherapy was superior to either monotherapy in the short term, and also superior to antidepressant alone but was as good as psychotherapy alone in the long term [6].

Benzodiazepines have been the most frequently prescribed medication for patients with panic disorder [7,8]. There are some advantages to treatment with benzodiazepines in that they lead to effects on panic attacks earlier [9] with milder adverse effects than antidepressants [10]. Nevertheless, the use of benzodiazepines has been associated with sedation, reduced coordination, cognitive impairments [11], increased accident proneness [12,13] and development of dependence [14], and it has been reported that a rebound of panic attacks can occur during taper [15].

Psychotherapy has also been reported to be effective in treating panic disorder [16-23] and is a potential alternative to the use of benzodiazepines without any adverse drug effects. On the other hand, psychotherapy has been said to need longer time to show its effects on panic disorder than benzodiazepines [9].

A benzodiazepine and psychotherapy are widely used together in practice. In a specialized clinic for cognitive-behaviour therapy, over three quarters of the patients were given benzodiazepines [24]. However, little evidence exists so far for any additional benefit to combination therapy and, if so, whether the benefit is worth the extra cost of combining two treatments [25-27]. Furthermore, some observational studies suggested that benzodiazepines actually interfered with cognitive-behavioral interventions [28-31], while others [32,33] suggested otherwise. We therefore need stronger evidence to get a more precise estimate of their efficacy and safety.

The primary objective of the present systematic review is to comprehensively search for and synthesize the best evidence on the combined psychotherapy plus benzodiazepines in comparison with either treatment alone for panic disorder with or without agoraphobia, in both the short- and long-term.

## Methods

### Inclusion criteria

All relevant randomised controlled trials comparing combined benzodiazepine and psychotherapy with either therapy alone in adult patients with panic disorder with or without agoraphobia, diagnosed according to operationalized criteria such as DSM-III, DSM-III-R, DSM-IV or ICD-10 were included. Patients with secondary mental disorders were included but the effect of this decision was to be examined in a sensitivity analysis.

For psychotherapies, individual or group forms of psychological treatments, such as cognitive therapy, behaviour therapy, cognitive-behaviour therapy and psychodynamic therapy were included regardless of their frequency and duration.

Our primary outcome both of short-term and long-term efficacy was "response" defined by global judgement, such as "very much" or "much" improved in Clinical Global Impressions – Improvement scale if the data were available. When global assessment data were not available, reduction from the baseline in phobic avoidance was used for response. We did not take account of the number or severity of panic attacks as our definition of response, because it was not panic but rather phobic avoidance that has been shown to correlate with the severity of panic disorder with agoraphobia [34].

We also examined global severity, frequency or severity of panic attacks, phobic avoidance, general anxiety, depression, social functioning, patients' satisfaction and cost effectiveness.

Adverse effects were evaluated by looking at the number of dropouts due to adverse effects, and total number of dropouts for any reason was examined as a proxy measure of treatment acceptability.

### Identification of trials

We electronically searched the Cochrane Collaboration Depression, Anxiety and Neurosis Group Registers (CCDAN REGISTERS) on October 11, 2005. This comprehensive register is updated regularly adding the results of searches of The Cochrane Library, MEDLINE (1966-), EMBASE (1980-), CINAHL (1982-), PsycINFO (1974-), PSYNDEX (1977-) and LILACS (1982–1999) and handsearches of major psychiatric, medical journals, conference proceedings and trial registers. Moreover, the register is being coded continuously with respect to characteristics of studies such as the kind of interventions and their concomitant use, by looking through the full article of relevant studies manually. The studies already coded are being stored in CCDANCTR-Studies and the others in CCDANCTR-References. The register contains more than 24,000 records on trials comparing treatment options within the scope of the CCDAN. One complementary search for additional relevant trials was conducted with The Cochrane Central Register of Controlled Trials (CENTRAL).

Three independent reviewers (NW, RC and TAF) examined titles and abstracts of studies identified by the electronic searches and then checked full articles for eligibility. To identify further trials, references of these selected studies and of other review papers were also checked, representative studies were subjected to SciSearch and experts in the field were contacted. No language restriction was imposed.

### Quality assessment and data extraction

Two independent reviewers (NW and TAF) assessed the methodological quality of the selected studies.

The criteria for quality assessment were based on recommendations in the Cochrane Handbook for Systematic Reviews of Interventions [35], which focused on the quality of allocation concealment. We also rated whether at least one outcome measure was assessed by an independent assessor blind to treatment allocation. In addition, regarding psychotherapy, its adequacy is considered to be essential for effective treatment, and to depend on the expertise of the practitioner and the therapist's adherence to a particular form of therapy. We assessed the adequacy of psychotherapy in each study and if the authors gave sufficiently detailed description of the therapy procedure, and if audiotapes of the psychotherapy process were examined by a third reviewer. The three reviewers independently extracted data from the original reports using data extraction forms. For studies where exact numbers of responders were not presented but only their means and standard deviations (SDs) of the global severity measure, we imputed response rates by using a validated imputation method [36] in order to conduct intention-to-treat (ITT) analyses as described below. Any disagreement was resolved by consensus between all three reviewers.

### Data synthesis

Data were double-entered into Review Manager 4.2 to check for accuracy. Meta-analyses were then performed.

For dichotomous outcomes, ITT analysis was adopted. When dropouts were excluded from any assessment in the primary studies (for example, those who never returned for assessment after randomisation), they were considered non-responders both in the combination group and monotherapy group. Relative risks (RR) and their 95% confidence intervals were calculated using random effects model rather than a fixed effects model because of its generalizability [37].

For continuous outcomes, the standardized weighted mean difference (SMD) was calculated for end-point data of completers only using random effects model.

Heterogeneity, which refers to variability among studies in a systematic review and generally derives from clinical, methodological or statistical diversity [35], was assessed by Chi-square statistics and I-square statistics [38]. When substantive heterogeneity was found (p < 0.10 or I-squared > 30%), sources were investigated.

We also planned sub-group analyses in terms of types of psychotherapy, and sensitivity analyses to examine robustness of the review's findings, by a) limiting to trials with patients who had no comorbid physical or mental disorders, b) excluding trials with no regular concomitant use of an antidepressant, c) limiting to trials where the psychotherapy used had high adequacy, and d) limiting to trials where both allocation concealment and assessor blindness were noted.

## Results

### Description of studies

The electronic search identified 12 studies from CCDANCTR-Studies, 123 from CCDANCTR-References and 67 from CENTRAL. Browsing their titles and abstracts, 60 articles were identified by either of the two independent reviewers as possible candidates and their full copies were obtained. Ten studies were then selected to proceed to strict eligibility check stage. During further reference search, SciSearch and personal contacts, one additional study possibly eligible was identified. Two independent reviewers examined the strict eligibility of these 11 studies and 4 studies remained. The inter-rater reliability of the items of strict eligibility criteria was percentage agreement of 96% and kappa of 0.71 (95%CI: 0.37 to 1.0). Discrepancies were resolved by consensus. Two of the studies were finally excluded because benzodiazepine was given only on the day of the visit [39] or because only the number of completers in both intervention and control groups were described [40] (Table 1), and we arrived at two studies [20,41] (Table 2).

**Table 1:** Reasons for excluding studies

**Study**	**Reason for exclusion**
Chambless et al. 1982 [59]	The combination arm used barbiturates, not benzodiazepines.
Chouinard et al. 1982 [60]	Some participants had combination therapy but their outcomes were not reported.
Craske 1991 [61]	The interventions did not involve psychotherapy combined with benzodiazepine.
Echeburua et al. 1993 [62]	Participants were diagnosed as agoraphobia but had no panic attack.
Hafner and Marks 1976 [39]	Participants did not take benzodiazepine on regular basis but on the day of visit only.
Johnston and Gath 1973 [63]	Participants did not take benzodiazepine on regular basis but on the day of flooding only.
Lopez-Alonso and Gomze-Jarabo 2000 [40]	Only the number of completers in both intervention and control groups were described.
Otto et al. 1993 [64]	All participants were prescribed benzodiazepine and this was then tapered.
Riley et al. 1995 [65]	The interventions did not involve the combination of psychotherapy and benzodiazepine.
Spiegel et al. 1994 [66]	All participants were prescribed benzodiazepine and this was then tapered.
Whitehead et al. 1978 [67]	All participants had animal phobia, not panic disorder.

**Table 2:** Characteristics of the included studies

**Study**	**Participants**	**Acute phase interventions**	**Maintenance interventions**	**Follow-up**	**Definition of response**	**Other outcomes**
**Marks 1993 [20]**	**Diagnosis: **DSM-III PD with AG **Mean duration**: 8 years **Mean age: **35 years 81% female **Psychiatric comorbidity: **10% current MDD, 30% past MDD, 10% social phobia, 25% specific phobia Physical comorbidity: unspecified	**Duration: **16 weeks Weeks 0–8: benzodiazepine (alprazolam [A]) or pill-placebo (P), with live exposure (E) or with relaxation (R) (R = psychological-placebo). Weeks 9–16: A & P tapered to zero, no E or R given. i.e. 4 groups: AR, AE, PR, PE AR=bz alone AE=bz+psychotherapy PR=double placebo PE=psychotherapy alone 1. Benzodiazepine mean dose 5.8 mg/day) 2. Psychotherapy or placebo = 2 hours live exposure or relaxation 6 sessions	None	Assessments "at the end of acute treatment" gathered at 2 weeks after actual treatment termination. Long-term follow-up at 7 months after treatment termination.	'Very much' or 'much' improved on Clinician's CGI. Numbers of responders calculated using normal curve [36]	**Global severity of PD: **7-point CGI rated by assessors **Panic attack**: total number of major panics per week **Agoraphobia: **4 phobic targets avoidance **General anxiety: **14-item HAM-A **Depression: **17-item HAM-D Social functioning: 9-point assessor-rated scale for work, social, and family adjustment
**Wardle 1994 [41]**	**Diagnosis: **ICD-9 and DSM-III-R AG **Mean duration: **11–12 years **Mean age: **41–45 years in each arm 82% female **Psychiatric co morbidity: **Not specified Excluded if had comorbid heart disease, alcohol problems, drug abuse, pregnancy or anticipated pregnancy, or major psychotic illness	**Duration: **16 weeks 16 weeks for benzodiazepine (with drug taper) and 8 weeks for psychotherapy (week 4–12) 1. Diazepam (the dose was kept to 5 mg/day unless the patient requested an increase) + BT (eight 2-hour sessions over a 7 week period by a clinical psychologists). Medication was tapered to zero from week 13 to 16. 2. BT + placebo drug	None	At 1, 6, 12 months after treatment discontinuation Patients were advised to continue self-exposure. After the acute treatment phase, all patients were withdrawn during 0–1 month after treatment discontinuation. No further treatment was offered over the follow-up period unless the patient's clinical condition necessitated intervention.	50% reduction on MAL from the baseline by looking at the raw data	**Global severity of panic disorder: **MAL **Panic attack: **Panic frequency in past week **Agoraphobia: **MAL **General anxiety: **STAIS-T **Depression: **BDI **Social functioning**: Not applicable

AG: agoraphobia; BDI: Beck Depression Inventory; BT: Behaviour therapy; CGI: Clinician's Global Improvement; HAM-A: Hamilton Anxiety Rating Scale; HAM-D: Hamilton Depression Rating Scale; MAL: Mobility Inventory 'Mobility Alone'; MDD: Major depressive disorder; MAL: Mobility index – Mobility alone; PD: panic disorder; STAIS-T: State-Trait Anxiety Inventory-Trait Anxiety

Marks and his colleagues provided two comparisons, which comprised of one comparison between combination therapy and psychotherapy alone and the other between combination and benzodiazepine alone [20,34,42-52]. Wardle and her colleagues provided a comparison between combination therapy and psychotherapy alone [41,53]. We therefore obtained data for a total of 243 participants from two studies (Table 3).

**Table 3:** Results of meta-analyses

			**Psychotherapy+Benzodiazepine vs Psychotherapy alone**			**Psychotherapy+Benzodiazepine vs Benzodiazepine alone**		
			
		**Statistical method**	**Number of comparisons**	**Number of participants**	**Effect size [95% CI]**	**Number of comparisons**	**Number of participants**	**Effect size [95% CI]**
**Acute phase treatment (at 2–4 months)**	Response	RR	2	166	1.25 [0.78, 2.03]	1	77	1.57 [0.83, 2.98]
	Global severity	SMD	2	122	0.15 [-0.21, 0.50]	1	68	0.63 [0.14, 1.11]
	Frequency of panic attacks	SMD	2	124	0.38 [0.02,0.74]	1	68	0.18 [-0.29, 0.66]
	Phobic avoidance	SMD	2	122	0.09 [-0.27, 0.45]	1	68	1.17 [0.65, 1.68]
	General anxiety	SMD	1	60	0.08 [-0.43, 0.59]	0	0	Not estimable
	Depression	SMD	2	120	0.17 [-0.23, 0.58]	1	68	0.14 [-0.34, 0.62]
	Social functioning	SMD	1	64	0.51 [0.01, 1.01]	1	68	1.05 [0.54, 1.56]
	Dropouts for any reason within 2–4 months	RR	2	166	0.81 [0.47, 1.38]	1	77	1.85 [0.50, 6.87]
	Dropouts due to side effects within 2–4 months	RR	0	0	Not estimable	0	0	Not estimable
**Immediately after the end of drug taper**	Response	RR	2	166	0.78 [0.45, 1.35]	1	77	3.39 [1.03, 11.21]
	Global/Avoidance/Panic	SMD	2	99	-0.31 [-0.71, 0.09]	1	53	0.75 [0.19, 1.31]
**6–12 months after treatment termination**	Response	RR	2	166	0.62 [0.36, 1.07]	1	77	2.31 [0.79, 6.74]
	Global/Avoidance/Panic	SMD	2	95	-0.19 [-0.59, 0.22]	1	35	0.99 [0.28, 1.70]

CI: confidence interval; RR: relative risk; SMD: standardized weighted mean difference

Regarding diagnostic criteria, the former study included patients who had panic disorder with agoraphobia, and the latter included patients with agoraphobia. Though the latter did not mention panic disorder in diagnostic inclusion criteria, contact with the authors revealed that all patients in the study had some panic attacks and almost all of the patients would be qualified as panic disorder with agoraphobia according to DSM-IV.

The former employed alprazolam as a benzodiazepine and exposure as psychotherapy; the latter did diazepam as a benzodiazepine and exposure as psychotherapy. The duration of interventions was 16 weeks in both studies, which included drug withdrawal (Table 2).

With regard to outcomes, means and standard errors (SEs) of clinical global rating were reported at all the time points of assessment in one study [49]. SDs were calculated from SEs and the numbers of responders ("very much or "much" improved on Clinician's Global Improvement) were imputed. The other study assessed clinical global rating only at the 12-months follow-up assessment [41], so we defined responders as patients with 50% reduction from baseline on the mobility when alone subscale (MAL) of the Mobility Inventory [54]. No study reported on patients' satisfaction and economic costs. The numbers of patients who dropped out due to side effects or suffered from withdrawal symptoms upon cessation of treatment were not specified either. No information is given about relapse rates during follow-up in both of the studies. Additional treatment (taking benzodiazepines after the treatment period) is reported in one study [41], but the number of participants in each of the intervention and the control arms is not given.

With regard to study quality, neither study described concealment of allocation but both reported having kept assessors blind. The inter-rater reliability of these two validity criteria was 100% agreement for both. Regarding adequacy of the psychotherapies provided, one study was rated high [20], and the other moderate [41].

### Psychotherapy plus Benzodiazepine versus Psychotherapy

#### Acute phase treatment

**Figure 1:**
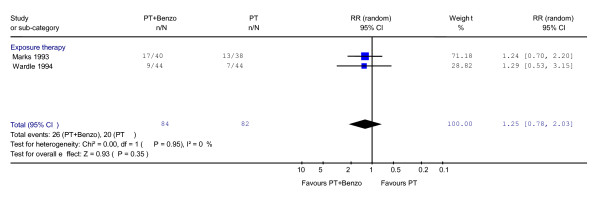
**Psychotherapy plus benzodiazepine vs. psychotherapy alone: response at 2–4 months during acute phase treatment**. Relative risk of response was calculated using both CGI [20] and Mobility Alone [41].

Two comparisons from two studies were included between combined psychotherapy and benzodiazepine versus psychotherapy alone in the acute phase treatment (Table 3). The data suggested the relative risk (RR) for response for the combination was 1.25 (95% CI: 0.78 to 2.03; P = 0.35) at 2 months during the acute phase treatment (Figure 1). No heterogeneity was observed (χ^2 ^= 0.00, df = 1, P = 0.95; I^2 ^= 0.0%).

**Figure 2:**
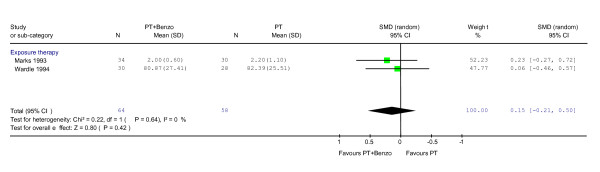
**Psychotherapy plus benzodiazepine vs. psychotherapy alone: global severity at 2–4 months during acute phase treatment**. Standardised mean difference was calculated using both CGI [20] and Mobility Alone [41].

With regard to secondary outcomes, although combination therapy was not shown to have significant superiority to psychotherapy alone in terms of global severity of panic disorder (Figure 2), the combination therapy was superior in panic frequency and social functioning (panic frequency: SMD 0.38; 95%CI 0.02 to 0.72; P = 0.04, social functioning: SMD 0.51; 95%CI 0.01 to 1.01; P = 0.05).

Both combination therapy and psychotherapy alone groups had similar dropout rates (RR = 0.81, 95% CI: 0.47 to 1.38; P = 0.44).

#### After the end of acute treatment

**Figure 3:**
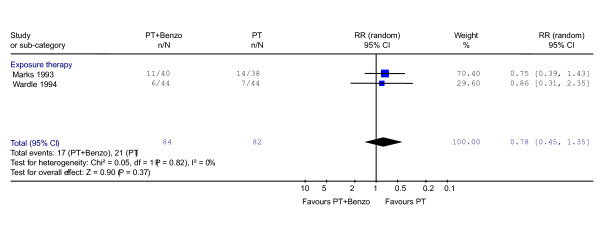
**Psychotherapy plus benzodiazepine vs. psychotherapy alone: response after the end of acute treatment**. Relative risk of response was calculated using both CGI [20] and Mobility Alone [41].

**Figure 4:**
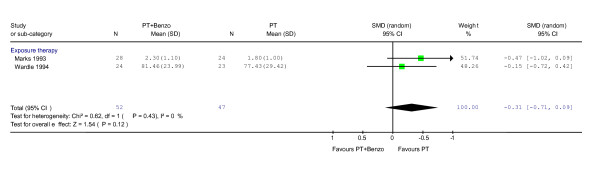
**Psychotherapy plus benzodiazepine vs. psychotherapy alone: global severity after the end of acute treatment**. Standardised mean difference was calculated using both CGI [20] and Mobility Alone [41].

Little difference between combination therapy and psychotherapy alone groups was shown on the whole (RR = 0.78, 95% CI: 0.45 to 1.35; P = 0.37) (Figure 3). No heterogeneity was observed (χ^2 ^= 0.05, df = 1, P = 0.82; I^2 ^= 0.0%). In terms of the global severity, weak trend of inferiority of the combination therapy was observed (SMD -0.31, -0.71 to 0.09, P = 0.12) (Figure 4).

#### Long-term after treatment termination

**Figure 5:**
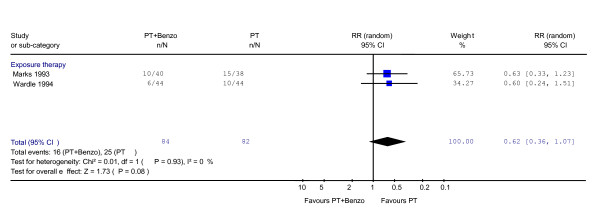
**Psychotherapy plus benzodiazepine vs. psychotherapy alone: response at 6–12 months after treatment termination**. Relative risk of response was calculated using both CGI [20] and Mobility Alone [41].

**Figure 6:**
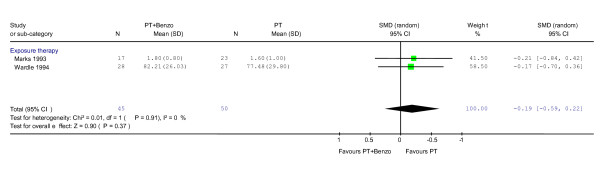
**Psychotherapy plus benzodiazepine vs. psychotherapy alone: global severity at 6–12 months after treatment termination**. Standardised mean difference was calculated using both CGI [20] and Mobility Alone [41].

At either 12 [41] or 7 [20] months of naturalistic follow-up after treatment termination, the RR of response rate showed a trend in favour of psychotherapy alone over combination (RR = 0.62, 95%CI: 0.36 to 1.07; P = 0.08) (Figure 5). No heterogeneity was observed (χ^2 ^= 0.01, df = 1, P = 0.93; I^2 ^= 0.0%). In terms of the global severity, this trend was not observed (SMD -0.19, -0.59 to 0.22, P = 0.37) (Figure 6).

### Psychotherapy plus Benzodiazepine versus Benzodiazepine treatment

#### Acute phase treatment

Comparing combined psychotherapy plus benzodiazepine with benzodiazepine alone in one comparison [20], the RR for response for the combination was 1.57 (95%CI: 0.83 to 2.98; P = 0.17) at 2 months during acute phase treatment (Table 3), which was not statistically significant.

The significant superiority of the combination therapy was observed for some secondary outcomes including global severity of the disorder (SMD 0.63; 95%CI 0.14 to 1.11), phobic avoidance (SMD 1.17; 0.65 to 1.68) and social functioning (SMD 1.80; 0.99 to 2.61). There was no significant difference between both arms in frequency of panic attacks and depression.

No difference was observed in the overall dropouts either.

#### After the end of acute phase treatment

Superiority of combination to benzodiazepine alone was observed both in the response rate (RR = 3.39, 95% CI: 1.03 to 11.21, P = 0.05) and the global severity (SMD = 0.75, 95% CI: 0.19 to 1.31, P = 0.009).

#### Long-term after treatment cessation

In terms of response after 7 months after treatment cessation, a weak trend in favour of the combination therapy (RR = 2.31, 95% CI: 0.79 to 6.74, P = 0.12) in terms of response rates, and a statistically significant superiority of the combination in terms of global severity was observed.

### Subgroup and sensitivity analyses

Due to the limited number of comparisons available, it was not possible to undertake any subgroup analyses or sensitivity analyses.

## Discussion

Despite extensive literature searches and personal communication, only two rigorous studies were identified that examined combined benzodiazepines and psychotherapy for panic disorder.

What little evidence there is suggests, however, that behaviour therapy (exposure) plus benzodiazepines may be superior to benzodiazepines alone, especially at the end of acute treatment (RR = 3.39, 1.03 to 11.21), and possibly also during the acute phase and at long-term follow-up. The combined therapy may also be superior to behaviour therapy alone during the acute phase, but this possible superiority may not persist and perhaps invert during the follow-up 6–12 months after treatment termination.

With respect to combination therapy versus benzodiazepines alone, secondary endpoints representing continuous measures also hint at the superiority of the combination during the acute phase treatment, in that the combination was significantly superior to benzodiazepines in terms of the global severity, phobic avoidance, and social functioning. Over the long term, this superiority was not statistically significant (RR = 2.31, 0.79 to 6.74). However, the secondary outcome demonstrated combination therapy was significantly superior to benzodiazepine alone. In addition, data from one of the included RCTs indicated that even placebo was not inferior to behaviour therapy alone or benzodiazepine alone for panic attacks as opposed to phobias and disability, for which placebo was indeed inferior. [20].

With regard to behaviour therapy alone, some secondary endpoints hinted at the superiority of the combination during the acute phase treatment (frequency of panic attack, social functioning). Some authors have argued that the combination therapy is superior to psychotherapy alone in the long term [28,31]. However the results in the present review failed to reach statistical significance in terms of response rate (RR = 0.62, 0.36 to 1.07, P = 0.08) or the secondary outcome representing global severity of panic attacks (SMD = -0.19, -0.59 to 0.22, P = 0.37).

Considering these results, benzodiazepine alone is not to be recommended for those who have access to appropriate resources of behaviour therapy.

We did not focus on the combined antidepressants and psychotherapy in the present review, but our companion systematic review on this topic also concluded that either combined therapy or psychotherapy alone rather than antidepressants alone might be chosen as first-line treatment for panic disorder with or without agoraphobia [6]. Combining the conclusions of the two reviews, pharmacotherapy alone, be it by an antidepressant or by a benzodiazepine, may be avoided in treatment for panic disorder where psychotherapy is available.

One has to note some possible weaknesses in the present review. First of all, only two studies including 243 patients diagnosed as panic disorder with agoraphobia were identified, type II errors were therefore likely and even the significant results might have limited generalizability. Moreover, we were not able to do any analysis on panic disorder without agoraphobia and managed only to establish very limited evidence for panic disorder with agoraphobia. Secondly, generalizability of the present findings beyond specialist psychiatric settings is not straightforward. The included studies were conducted at psychiatric hospitals under psychiatrists' and clinical psychologists' administration [20,41], so that generalizability of the results of the present review to primary care may be questioned. Another concern on generalizability of the review may arise from the fact that participants were limited to those having been able to tolerate being off medication before entering the study in one study [20]. In addition, regarding use of benzodiazepines, one included study employed a huge daily dose of alprazolam in both the benzodiazepine arms [20] compared to the dose usually recommended in clinical practice, and this could have had negative effects on these arms. The other included study [41] used diazepam as benzodiazepine in relatively lower dose compared to previous studies which involved diazepam as treatment for panic disorder [55-58]. Although diazepam has been demonstrated not only superior to pill-placebo but also equally effective compared with alprazolam [56,58], this issue could have diminished efficacy of diazepam and have diluted differences between combined therapy and psychotherapy alone. Thirdly, the definition of "response" employed in the present study differs from study to study, due to reported outcomes in the original reports. Clinical global rating was employed in one study [20] and phobic avoidance scale in the other [41]. The proportions of responders are much smaller in one study [41] than the other [20], possibly due to different definitions of response. Although no significant heterogeneity has been observed through the analyses in the present review, one might think these definitions are arbitrary or plausible. However, we failed to obtain clinical global scale in the latter [41] in spite of contacting the original authors. We therefore utilized only phobic avoidance scale to define the response, since this has been shown to better represent the patients' global status [34]. Last but not least, all the included studies dealt with psychotherapy in the form of exposure therapy. Other forms of psychotherapies including cognitive restructuring and psychodynamic therapy also need to be examined in combination with benzodiazepines.

On the other hand, the present review may have several strengths. First, we performed a systematic and comprehensive search for the relevant trials. All references to possible candidate studies were investigated to identify relevant ones. The authors of the included studies were contacted to give more information about their studies and other possibly relevant ones. Consequently, our analyses managed to include some unpublished data (MAL) in one study [41]. Second, though there are many narrative reviews on efficacy of combination therapy against either monotherapy that included both high-quality and low-quality studies all together, we limited our systematic review only to high quality RCTs. These RCTs were also conducted while keeping assessors blind. Our approach minimized bias and therefore offered more rigorous evidence. Third, we applied strict ITT principle, by means of imputing response rates from continuous measures of global severity for panic disorder and counting all the dropouts as non-responders, as we were interested in the fate of all patients who began the acute phase after randomisation, not just those who completed it. Counting dropouts as non-responders might appear too stringent as some dropouts may have improved, but we cannot confirm their percentage. Given this circumstance, this approach is the most conservative estimate of the response rate, and in the absence of any stronger and more rational alternative, we decided to remain conservative with regard to either treatment arm.

## Conclusion

In conclusion, we would like to make some recommendations both for clinicians and for researchers as follows.

### Implications for practice

Currently, there is inadequate evidence to assess the clinical effects of combined benzodiazepine and psychotherapy treatment for patients who are diagnosed as panic disorder with agoraphobia, and no rigorous RCT exists for panic disorder without agoraphobia or for psychotherapy other than exposure therapy. Based on this limited evidence, exposure therapy may be recommended for patients with agoraphobia who have access to appropriate resources. Benzodiazepine alone is not to be recommended for those in such a situation. It seems possible that combined therapy is superior to exposure therapy alone in the acute treatment, but after the acute treatment is terminated, this trend may be reversed.

### Implications for research

More high-quality trials for combined benzodiazepine and psychotherapy treatment must be conducted before making any stronger treatment recommendation.

## Competing interests

TAF has received several research grants and fees for speaking from a pharmaceutical company, which markets a benzodiazepine (ethyl loflazepate). RC and NW have no conflict of interest to declare.

## Authors' contributions

NW contributed to the concept and design, the development of the review protocol, the literature search, the selection of trials, the quality assessments of the trials, the data extraction, inputting the data to the statistical software, the data analysis, and wrote the first draft of the manuscript.

RC contributed to the development of the review protocol, the selection of trials, and the data extraction.

TAF contributed to the development of the review protocol, the literature search, the selection of trials, the quality assessments of the trials, and the data analysis.

All authors contributed to the interpretation of the results, critically revised and approved the final manuscript.

## Pre-publication history

The pre-publication history for this paper can be accessed here:


